# Evolving sex-specific trends in mental health-related emergency department visits (2010–2023): insights from 643 French general hospitals

**DOI:** 10.3389/fpubh.2025.1607649

**Published:** 2025-07-28

**Authors:** Guillaume Barbalat, Nicolas Franck

**Affiliations:** Centre Ressource de Réhabilitation Psychosociale et de Remédiation Cognitive, Hôpital Le Vinatier, Pôle Centre Rive Gauche, UMR 5229, CNRS & Université Claude Bernard Lyon 1, Lyon, France

**Keywords:** emergency department, affective disorders, non-affective psychotic disorders, substance use disorders, sex differences, temporal trends, general hospitals

## Abstract

**Background:**

Psychiatric disorders account for a significant proportion of emergency department (ED) visits, with notable sex-specific differences. However, how these disparities have evolved over time, particularly following the COVID-19 pandemic, remains poorly understood.

**Methods:**

We analyzed yearly ED visit data from 2010 to 2023 for individuals aged 18–65 with diagnoses of substance use, affective, and non-affective psychotic disorders from 643 French general hospitals. Fixed-effects models were used to examine sex-specific trends, with 2010 as the reference year for baseline analyses and 2019 for pandemic-era comparisons.

**Results:**

The mean rate of mental health-related ED visits was 6.8% during the study period. Compared to females, males exhibited a significant increase in ED visits related to affective and non-affective psychotic disorders since 2010. Compared to females, males showed significant reductions in substance use disorder visits post-pandemic (2021–2023 vs. 2019). Affective disorder visits among males transiently decreased in 2022.

**Conclusion:**

Our findings highlight evolving sex-specific trends in mental health-related ED visits, with males experiencing increases since 2010, and females facing disproportionate post-COVID-19 burdens. These findings can guide sex-specific healthcare resource allocation and enhance the delivery of mental health services.

## Introduction

Psychiatric disorders represent a significant and growing proportion of emergency department (ED) visits. In the UK, a 2021 report indicated that 5% of all hospital ED attendances in England were primarily due to mental ill-health (1). In the US, data from the National Hospital Ambulatory Medical Care Survey revealed that between 2007 and 2016, 8.3% of ED visits were associated with a psychiatric or substance use diagnosis (2). Mental health (MH)-related visits rose from 4.9 to 6.3% of all US ED visits between 1992 and 2001, with substance-related disorders, mood disorders, and anxiety disorders being the most prevalent diagnoses (3). More recent data indicate that this trend may be increasing, with MH-related ED visits constituting approximately 12.3% of all adult ED visits in 2017–2019 (4).

The rising utilization of EDs for psychiatric care poses critical challenges to healthcare systems. The increasing prevalence of mental disorders has led to longer wait times and reduced capacity in ED. For general hospitals, this trend leads to a specific challenge, increasing the complexity of care required, as ED staff often lacks specialized training to effectively manage and treat patients with mental health conditions. Strategies to address these challenges may include improving access to outpatient mental health services or enhancing early intervention programs.

Targeting subpopulations with disproportionately increased ED presentations can be another effective strategy. For instance, tailoring mental health practices to address the unique needs of women may help reduce their suffering (5) and, consequently, their ED presentations. Significant sex disparities in MH-related ED utilization have been observed, with men more frequently presenting for substance use disorders, and women more often seeking care for anxiety and depressive disorders (6). Sex-specific differences in mental disorders may be related to gendered social roles and power differentials (7). These differences can be shaped by disparities in access to resources, caregiving responsibilities, and exposure to stressors such as economic hardship or domestic violence. Additionally, societal expectations and gender norms may influence how mental health symptoms are expressed and reported, potentially affecting diagnosis and treatment outcomes.

The COVID-19 pandemic appears to have shifted established mental health trends in several ways. First, multiple studies have documented a general decrease in MH-related ED visits across various countries (8–12). However, some reports have noted absolute or relative surges in these visits (13, 14), which might result from new-onset (vs. already existing) diagnoses (15), highlighting the complexity of pandemic-related changes.

Additionally, emerging research emphasizes the importance of analyzing distinct time periods—such as pre-pandemic, acute pandemic, and post-restriction phases (16) to more accurately assess specific trends in MH-related ED utilization. Second, the pandemic seems to have exacerbated declines in well-being among women. Research indicates that female gender was associated with higher rates of depression, anxiety, and insomnia, as well as lower overall well-being during lockdown periods compared to males (17–20), although these findings were not entirely consistent across all studies (21).

These findings underscore the importance of considering temporal dynamics in sex-related mental health trends. To our knowledge, however, no studies have systematically examined these patterns over extended periods (e.g., multiple years). Examining these trends could offer valuable insights into shifting societal attitudes and emerging stressors that impact mental health differently across genders. Such insights may help guide policies to reduce mental health inequities and alleviate the growing burden of psychiatric care on ED. Using data from ED visits in general hospitals across France, this study aimed to examine the impact of sex on MH-related ED visits over a 14-year period (2010–2023).

## Methods

### Data

We obtained ED visit data from the “Organisation de la Surveillance Coordonnée des Urgences” (OSCoUr; “Organization of Coordinated Emergency Surveillance” established in 2004 following the 2003 heat wave in France). OSCoUr is a key component of the French syndromic surveillance system SurSaUD^®^ (“Surveillance Sanitaire des Urgences et des Décès”), which main objectives are to collect and analyze ED visit data in general hospitals across France; detect and monitor various public health events; evaluate the impact of known and unexpected events on population health. As of 2010, OSCoUr covered 40.1% of ED visits in France, reaching 76.8% in 2014 and 94.4% in 2018.

We used data for individuals aged 18–65, and the following three diagnosis categories:

- Substance use disorders: mental and behavioral disorders due to psychoactive substance use such as alcohol, opioids, cannabis, sedatives, cocaine, etc. [ICD10: F10-F19 (22)];
- Affective disorders: bipolar, major depressive, and other mood disorders [ICD10: F30-F39 (22)], as well as anxiety, dissociative, stress-related, and somatoform disorders [ICD10: F40-F49 (22)]. Notably, nicotine dependence (tobacco use disorder in DSM-5) was not excluded from our analysis, as our aim was to examine the full spectrum of substance use disorders;
- Non-affective psychotic disorders: schizophrenia, delusional, and other non-mood psychotic disorders [ICD10: F10-F19 (22)].

For each hospital, annual counts of ED visits were collected by sex (male vs. female) and diagnosis category for the years 2010 to 2023. MH-related ED visits were defined as any ED presentation with a principal or associated mental health diagnosis. In other words, the chief complaint could be either a mental health issue or a somatic issue, as long as a mental health diagnosis was recorded as primary or secondary. Observations in which the number of ED visits per year was fewer than five were excluded from the analysis. For each diagnosis category, the reported value reflects the total number of cases within that specific category. Because a single patient may have more than one mental health diagnosis across different categories, it is possible for some patients to be counted in multiple categories in the raw data. Finally, we did not limit our analysis to a fixed set of hospitals throughout the study period, considering the whole set of available data (hospitals that later ceased operations as well as new hospitals). Overall, the number of hospitals included in the analysis increased over time from *N* = 263 (year 2010) to *N* = 643 (year 2023).

### Analysis

For each diagnosis category, we employed a linear model using the log-transformed rate of ED visits (ED count scaled by the total number of ED visits) as the dependent variable. Our primary focus was on the interaction between sex (male vs. female) and year (14 levels from 2010 to 2023), taking 2010 as the reference year. The model also included sex and the log-transformed total ED counts as covariates, with the latter serving to account for the effect of patient load. To control for potential confounding factors related to temporal and geographical effects that might bias our estimates of sex differences over time, we incorporated both year and hospital fixed effects into the models. Indeed, we reasoned that the temporal influence of sex on ED visits may be related to both time-varying features (e.g., economic downturns) and time-invariant hospital characteristics (e.g., geographic barriers; population density variations within the catchment area; travel times and accessibility; hospital ownership status; specific health conditions treated at each hospital).

To assess whether COVID-19 significantly altered sex-specific trends in MH-related ED visits, we conducted a secondary analysis using 2019 as the reference year. In this framework, coefficients for pandemic (2020) and post-pandemic (2021–2023) years represent deviations in MH-related ED visit rates relative to pre-pandemic (2019) levels, enabling males vs. females comparison of acute and longer-term pandemic effects.

To account for the correlation of observations within hospitals, we employed cluster-robust standard errors. We opted not to use year-clustering due to the relatively small number of years (<30), as this could potentially lead to unreliable inference resulting from downward-biased standard errors. We conducted this analysis using the *fixest* package in R.

## Results

The number of hospitals included in the analysis was *N* = 609 for substance use disorders (11,307 observations), *N* = 568 for affective disorders (10,194 observations), and *N* = 482 for non-affective psychotic disorders (7,865 observations). The total number of recorded ED visits increased steadily from 2,806,094 in 2010 to 8,822,766 in 2023, with a temporary decline to 6,933,911 in 2020.

Table 1 reports yearly MH-related ED visit rates stratified by mental health condition and sex. Over the study period, the mean rate of MH-related ED visits was 2.5% for substance use disorders (2010: 3.3%; 2023: 1.7%); 3.3% for affective disorders (2010: 3.9%; 2023: 2.9%); and 0.6% for non-affective psychotic disorders (2010: 0.7%; 2023: 0.6%). The three diagnoses yield an aggregated mean rate of MH-related ED visits of 6.4% (2010: 7.9%; 2023: 5.2%).

**Table 1:** Yearly MH-related ED visit rates stratified by mental health condition and sex.

Year	Affective disorders				Non-affective psychotic disorders				Substance use disorders			
	Female		Male		Female		Male		Female		Male	
	Mean	SD	Mean	SD	Mean	SD	Mean	SD	Mean	SD	Mean	SD
2010	0.049	0.034	0.028	0.021	0.006	0.009	0.007	0.012	0.021	0.021	0.044	0.040
2011	0.047	0.032	0.027	0.019	0.006	0.008	0.007	0.012	0.022	0.023	0.046	0.043
2012	0.048	0.042	0.027	0.026	0.006	0.013	0.007	0.020	0.020	0.020	0.042	0.039
2013	0.044	0.043	0.027	0.029	0.006	0.016	0.008	0.025	0.017	0.014	0.037	0.033
2014	0.042	0.034	0.027	0.022	0.005	0.011	0.007	0.017	0.016	0.015	0.035	0.032
2015	0.043	0.040	0.027	0.026	0.005	0.013	0.007	0.020	0.016	0.022	0.035	0.041
2016	0.040	0.034	0.026	0.021	0.005	0.011	0.007	0.018	0.016	0.027	0.033	0.043
2017	0.040	0.033	0.025	0.020	0.005	0.012	0.007	0.019	0.016	0.028	0.032	0.043
2018	0.038	0.029	0.024	0.015	0.005	0.008	0.006	0.012	0.015	0.026	0.030	0.035
2019	0.036	0.030	0.024	0.020	0.005	0.013	0.007	0.020	0.013	0.014	0.027	0.025
2020	0.038	0.030	0.025	0.020	0.005	0.013	0.007	0.021	0.013	0.013	0.027	0.022
2021	0.036	0.026	0.023	0.016	0.004	0.006	0.006	0.009	0.012	0.012	0.024	0.018
2022	0.036	0.040	0.023	0.027	0.004	0.006	0.007	0.014	0.012	0.012	0.023	0.022
2023	0.035	0.034	0.023	0.023	0.004	0.005	0.007	0.013	0.012	0.013	0.022	0.019
Hospital-level variation^a^	0.031				0.012				0.025			

MH, mental health; ED, emergency department.

a Standard deviation of ED visit rates among hospitals. Note the substantial variation among hospitals.

We then modeled the log-rate of MH-related ED visits as a linear function of sex, the log-total ED count, and the interaction between sex and years, including years and hospitals as fixed effects. The adjusted *R*^2^ was 0.83 for the substance use disorders model; 0.81 for the affective disorders model; and 0.75 for the non-affective psychotic disorders model. The proportion of variance explained within hospitals was substantial (*R*^2^ = 0.51, 0.21, and 0.47, respectively). The variation of hospital-specific parameter estimates was also substantial (Tables 2, 3).

**Table 2:** Parameter estimates of the fixed effects models using 2010 as the reference year.

Year	Affective disorders			Non-affective psychotic disorders			Substance use disorders		
	Mean estimate	Std error	*p* value	Mean estimate	Std error	*p* value	Mean estimate	Std error	*p* value
Sex	−0.551	0.018	<0.001	0.283	0.033	<0.001	0.843	0.027	<0.001
Total	−0.153	0.028	<0.001	−0.424	0.055	<0.001	−0.154	0.026	<0.001
Male by year (ref female-2010)									
2011	0.016	0.019	0.389	−0.008	0.038	0.841	0.015	0.027	0.582
2012	0.011	0.020	0.572	0.035	0.038	0.353	−0.030	0.032	0.339
2013	0.050	0.018	0.005	0.065	0.040	0.108	−0.009	0.029	0.770
2014	0.079	0.020	<0.001	0.048	0.035	0.173	0.037	0.030	0.219
2015	0.075	0.019	<0.001	0.050	0.037	0.172	0.044	0.029	0.139
2016	0.093	0.019	<0.001	0.113	0.038	0.003	0.026	0.029	0.374
2017	0.104	0.019	<0.001	0.116	0.036	0.002	0.011	0.031	0.734
2018	0.110	0.019	<0.001	0.123	0.037	0.001	0.034	0.032	0.282
2019	0.144	0.020	<0.001	0.167	0.037	<0.001	0.012	0.031	0.703
2020	0.157	0.019	<0.001	0.126	0.037	0.001	0.006	0.033	0.858
2021	0.123	0.019	<0.001	0.152	0.036	<0.001	−0.038	0.032	0.235
2022	0.114	0.019	<0.001	0.153	0.038	<0.001	−0.064	0.033	0.056
2023	0.121	0.020	<0.001	0.122	0.036	0.001	−0.044	0.032	0.176
Hospital-level variation^a^	0.994			0.865			1.08		

Std error, standard error.

a Standard deviation of hospital-specific parameter estimates. Note the substantial variation among hospitals.

**Table 3:** Parameter estimates of the fixed effects models using 2019 as the reference year.

Year	Affective disorders			Non-affective psychotic disorders			Substance use disorders		
	Mean estimate	Std error	*p* value	Mean estimate	Std error	*p* value	Mean estimate	Std error	*p* value
Sex	−0.407	0.012	<0.001	0.450	0.023	<0.001	0.855	0.020	<0.001
Total	−0.153	0.028	<0.001	−0.424	0.055	<0.001	−0.154	0.026	<0.001
Male by year (ref female-2019)									
2010	−0.144	0.020	<0.001	−0.167	0.037	<0.001	−0.012	0.031	0.703
2011	−0.128	0.019	<0.001	−0.175	0.035	<0.001	0.003	0.030	0.919
2012	−0.133	0.018	<0.001	−0.132	0.032	<0.001	−0.042	0.029	0.150
2013	−0.094	0.015	<0.001	−0.102	0.032	0.001	−0.021	0.026	0.435
2014	−0.065	0.016	<0.001	−0.119	0.031	<0.001	0.025	0.026	0.331
2015	−0.069	0.016	<0.001	−0.117	0.032	<0.001	0.032	0.023	0.168
2016	−0.051	0.015	0.001	−0.054	0.028	0.054	0.014	0.022	0.528
2017	−0.040	0.014	0.004	−0.051	0.027	0.057	−0.001	0.021	0.945
2018	−0.034	0.013	0.011	−0.044	0.028	0.118	0.022	0.020	0.266
2020	0.013	0.012	0.298	−0.041	0.026	0.121	−0.006	0.019	0.743
2021	−0.021	0.013	0.096	−0.015	0.027	0.588	−0.050	0.020	0.011
2022	−0.030	0.013	0.018	−0.014	0.027	0.594	−0.076	0.021	<0.001
2023	−0.023	0.014	0.092	−0.045	0.027	0.100	−0.056	0.022	0.010
Hospital-level variation^a^	0.994			0.865			1.08		

Std error, standard error.

a Standard deviation of hospital-specific parameter estimates. Note the substantial variation among hospitals.

There was a significant negative effect of the total number of ED visits on the rate of MH-related ED visits for each of the three diagnosis categories (all *p*’s < 0.001). In addition, male sex was associated with an increased rate of ED visits related to substance use and non-affective psychotic disorders (mean coefficients: 0.84 and 0.28, respectively; both *p*’s < 0.001), but with a decreased rate of ED visits related to affective disorders (mean coefficient: −0.55; *p* < 0.001).

**Figure 1:**
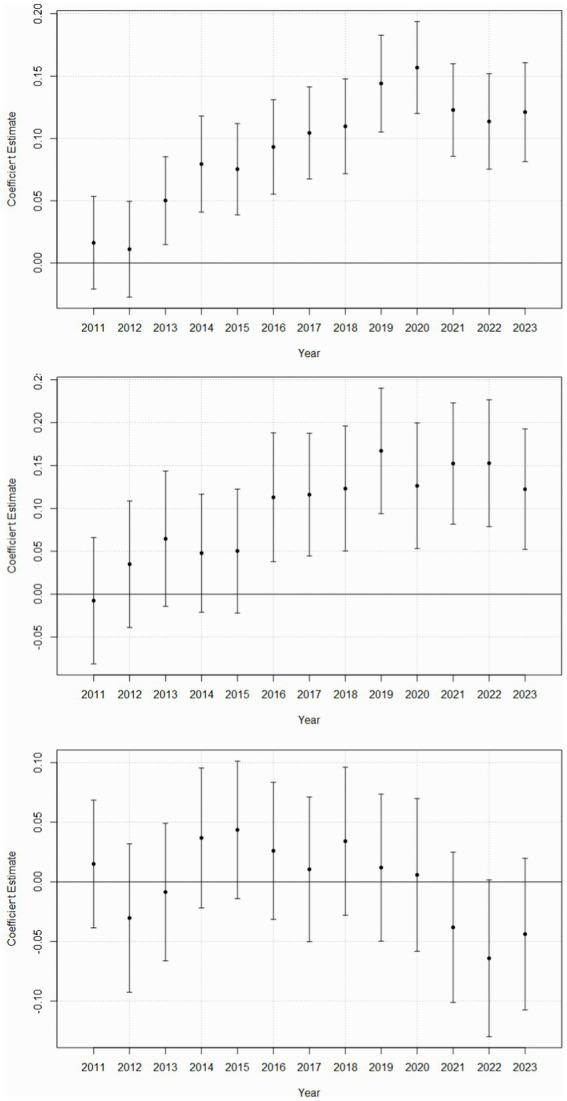
Sex-specific temporal trends in MH-related ED visits. MH, mental health; ED, emergency department. Coefficient estimates and 95% confidence intervals are plotted for each year, with year 2010 as the reference year and female sex as the reference group. A positive coefficient for a specific year indicates that, compared to females in 2010, males in that year had a higher rate of MH-related ED visits. Conversely, a negative coefficient for a specific year indicates that, compared to females in 2010, males in that year had a lower rate of MH-related ED visits. Confidence intervals were calculated using methods that account for within-hospital correlation of observations, ensuring proper adjustment for clustering effects inherent to hospital-level care delivery data. Confidence intervals that include zero are not significant. Upper panel. Affective disorders. Middle panel. Non-affective psychotic disorders Lower panel. Substance use disorders.

Compared to females, males saw a progressive increase of ED visits related to affective disorders since 2010, with significant differences observed from 2013 onwards (mean coefficients from 2013: all *b*’s > 0.05, all *p*’s < 0.01; Table 2; Figure 1). Similarly, males saw a progressive increase of ED visit related to non-affective psychotic disorders, with significant differences observed from 2016 onwards (mean coefficients from 2016: all *b*’s > 0.11, all *p*’s < 0.01; Table 2; Figure 1). Such temporal trend was not observed for substance use disorders (Table 2; Figure 1).

In addition, compared to females, males experienced significant reductions in substance use disorder-related ED visits in 2021–2023 vs. 2019 (all *b*’s < −0.05, all *p*’s < 0.01; Table 3). However, there were no sex-specific differences in affective and non-affective psychotic disorder-related visits post-pandemic, except for a transient decrease in affective disorder visits among males in 2022 (*b* = −0.03, *p* = 0.02; Table 3).

## Discussion

Consistent with previous research, we found that ED visit rates were generally higher among females for affective (mood and anxiety) disorders (6), while lower for substance use and non-affective psychotic disorders. Our results nevertheless challenge the widely held notion that women are faster than men to consult doctors. Indeed, since 2010, men have experienced a greater increase in ED visit rates for affective and non-affective psychotic disorders compared to women, while no such increase was observed for substance use disorders.

Fluctuations in ED visits may arise from two main phenomena: changes in the incidence of new-onset disorders, or changes in the severity of existing disorders. For example, a recent German study found a marked increase in new-onset psychiatric diagnoses during the COVID-19 pandemic, specifically for substance-related and addictive disorders, depressive disorders, schizophrenia spectrum and psychotic disorders, and anxiety disorders (15). While our study is unable to determine whether changes in ED visits over time are due to an increase in new-onset disorders or to changes in the severity of existing disorders, distinguishing between these factors would be crucial for understanding the underlying causes of such surges and for designing effective interventions. Specifically, a rise in new-onset cases suggests an increase in disease incidence within the population, which calls for screening programs or preventive measures targeting environmental or behavioral risk factors. In contrast, increased severity of existing disorders indicates that patients with known conditions are presenting with more acute or complicated symptoms, potentially due to gaps in outpatient management—such as care coordination, access to primary care, or enhanced community support—or to changes in disease progression. Additionally, external factors like job loss and diminished social relationships, as seen during the COVID-19 period, may further contribute to this trend (15).

Typically, men have been diagnosed with fewer cases of depression and anxiety disorders, despite their suicide rate being four times higher than that of women (23). However, over the past decade, evidence indicates a decline in mental well-being among men. For instance, a long-term study revealed that the prevalence of mental distress among men aged 16–24 increased from approximately 14% in 1991 to 19% by 2018 (24). Additionally, a report by Mind, a UK mental health charity, noted that as of 2019, about 40% men regularly feel worried or low, a figure that rose by 6% since 2009 (25). Such a decline in men’s mental health has been observed at twice the rate of that among women, even though more women than men regularly report feeling worried or low (25). Additionally, a 2018 UK well-being survey revealed that men report lower average life satisfaction compared to women (26).

The increase in MH-related ED visits among men over the past decade likely stems from a combination of factors. Economic pressures, such as financial strain and shifting labor markets, challenge traditional notions of masculinity tied to economic contribution (27). Societal expectations for men to appear strong, competitive and self-reliant, combined with pressures from social media comparisons (28), may further erode self-worth. Men’s tendency to avoid seeking mental health care—often influenced by stigma and the association of help-seeking with weakness or femininity (29)—has historically exacerbated the problem. However, there appears to be a growing openness around men’s emotional well-being and an increasing recognition of mental health as vital (30), which may be encouraging more disclosure and signaling a positive cultural shift toward addressing these challenges.

When using 2019 (the last pre-COVID year) as the reference year, we observed reduced rates of MH-related ED visits in males compared to females for substance use disorders from 2021 onward, with a similar pattern emerging for affective disorders specifically in 2022. Recent evidence indicates that while males continue to exhibit higher absolute rates of substance use disorders, the relative increase in substance use-related ED visits post-2020 was disproportionately observed among females, particularly in youth and young adult populations (31–34). The post-2020 timing of these disparities suggests they reflect systemic failures rather than acute pandemic stressors. Women’s over-representation in industries hardest hit by prolonged pandemic disruptions—including healthcare, hospitality, and education—likely exacerbated existing inequities. These sectors faced slow recovery trajectories (35) and increased workplace stigmatization (36), potentially intensifying psychological distress among female populations.

Strengths of this study include its large number of observations, its relatively extensive temporal span, and its consideration of hospital heterogeneity. However, the study is not without limitations. First, the analysis relied on hospital-level data rather than individual-level data. Second, we were unable to distinguish between principal and associated diagnoses. However, including both categories ensured that our findings reflect mental health diagnoses as contributing to at least some aspect of the reason for ED presentation. Third, sex-specific trends may differ among various substance use disorders (e.g., alcohol vs. tobacco vs. opioids, etc.). For example, a general decline in smoking rates, paired with a relative increase among women compared to men, may account for the pattern we observe in substance use disorders. On the other hand, some disorders, such as tobacco addiction, may not always be systematically diagnosed, which could introduce inter-rater bias. However, most disorders (apart from alcohol and tobacco) would likely have too few cases to be analyzed independently; moreover, the original dataset was aggregated by diagnosis group, which precluded subgroup analyses. Nevertheless, it would be valuable for future research to investigate sex and temporal trends for individual substance use disorders, potentially using individual-level data. Fourth, the study did not include sociodemographic data such as race, ethnicity or age groups. Nonetheless, the use of hospital and year fixed effects, combined with the inclusion of the total number of ED visits as a covariate, helped account for some variation associated with sociodemographic factors linked to temporal and geographical differences. Fifth, our dataset only included French general hospitals. As a result, the findings may not be generalizable to ED visits in psychiatric hospitals, and to healthcare systems outside France. Overall, we consider this analysis largely exploratory and hope it provides foundational data to stimulate further sex-specific research on MH-related ED presentations.

In conclusion, using data from a large sample of French general hospitals, we observed a temporal increase in ED visits related to affective and non-affective psychotic disorders among males compared to females since 2010. We also observed increases in ED visits related to substance use and affective disorders among females in the post-COVID-19 period. Further research is needed to better understand the unique mental health challenges faced by both males and females. Understanding such sex-specific trends could provide valuable evidence for healthcare systems to justify allocating more resources toward mental health. If the observed increase in ED visits related to affective and non-affective psychotic disorders among males is confirmed and is linked to challenges to masculinity and erosion of self-worth, it may be valuable to consider campaigns aimed at improving men’s access to mental health services and implementing programs that address self-worth in relation to masculine identity. Similarly, rising ED presentations related to substance use disorders among women could be addressed by strengthening social support – particularly for caregiving and workplace responsibilities – and by reducing resource inequalities. Future research should prioritize investigating the effectiveness of these interventions.

## Data Availability

The data analyzed in this study is subject to the following licenses/restrictions: The corresponding author has entered into an agreement with the data provider, which requires the deletion of the data upon completion of all analyses. As a result, the data supporting the findings of this study cannot be made publicly available. However, further inquiries regarding the data used in this study may be directed to the data provider. Requests to access these datasets should be directed to guillaumebarbalat@gmail.com.
